# Evaluation of Three Point-of-Care Tests for Detection of *Toxoplasma* Immunoglobulin IgG and IgM in the United States: Proof of Concept and Challenges

**DOI:** 10.1093/ofid/ofy215

**Published:** 2018-10-29

**Authors:** Carlos A Gomez, Laura N Budvytyte, Cindy Press, Lily Zhou, Rima McLeod, Yvonne Maldonado, Jose G Montoya, Despina G Contopoulos-Ioannidis

**Affiliations:** 1 Division of Infectious Diseases and Geographic Medicine, Department of Medicine, Stanford University School of Medicine, California; 2 Department of Pediatrics, Division of Infectious Diseases, Stanford University School of Medicine, California; 3 Palo Alto Medical Foundation Toxoplasma Serology Laboratory, National Reference Center for the Study and Diagnosis of Toxoplasmosis, California; 4 Toxoplasmosis Center, Department of Pediatrics, Division of Infectious Diseases, Ophthalmology and Visual Sciences, University of Chicago, Illinois

**Keywords:** immunoglobulin G, immunoglobulin M, *Toxoplasma gondii*, toxoplasmosis, point-of-care testing

## Abstract

**Background:**

The cost of conventional serological testing for toxoplasmosis discourages universal adoption of prenatal monthly screening programs to prevent congenital toxoplasmosis. Point-of-care (POC) technology may constitute a cost-effective approach.

**Methods:**

We evaluated the diagnostic accuracy of 3 *Toxoplasma* POC tests against gold-standard testing performed at Palo Alto Medical Foundation *Toxoplasma* Serology Laboratory (PAMF-TSL). The POC tests included the following: Toxo IgG/IgM Rapid Test (Biopanda) and the OnSite Toxo IgG/IgM Combo-Rapid-*test* that detect IgG and IgM separately, and the Toxoplasma ICT-IgG-IgM-*bk* (LDBIO) that detects either or both immunoglobulin IgG/IgM in combination. Samples were selected from PAMF-TSL biobank (n = 210) and Centers for Disease Control and Prevention *Toxoplasma* 1998 Human Serum Panel (n = 100). Based on PAMF-TSL testing, *Toxoplasma*-infection status was classified in 4 categories: acute infections (n = 85), chronic infections (n = 85), false-positive *Toxoplasma* IgM (n = 60), and seronegative (n = 80). The POC testing was performed in duplicate following manufacturer’s instructions by investigators blinded to PAMF-TSL results. Sensitivity and specificity were calculated.

**Results:**

A total of 1860 POC tests were performed. For detection of *Toxoplasma* IgG, sensitivity was 100% (170 of 170; 95% confidence interval [CI], 97.8%–100%) for all 3 POC kits; specificity was also comparable at 96.3% (77 of 80; 95% CI, 89.5%–98.9%), 97.5% (78 of 80; 95% CI, 91.3%–99.6%), and 98.8% (79 of 80; 95% CI, 93.2%–99.9%). However, sensitivity for detection of *Toxoplasma* IgM varied significantly across POC tests: Biopanda, 62.2% (51 of 82; 95% CI, 51.4%–71.9%); OnSite, 28% (23 of 82; 95% CI, 19.5%–38.6%); and LDBIO combined IgG/IgM, 100% (82 of 82; 95% CI, 95.5%–100%). Diagnostic accuracy was significantly higher for the LDBIO POC kit. The POC kits did not exhibit cross-reactivity for false-positive *Toxoplasma*-IgM sera.

**Conclusions:**

The 3 evaluated POC kits revealed optimal sensitivity for *Toxoplasma*-IgG antibodies. The *LDBIO*-POC test exhibited 100% sensitivity for the combined detection of IgG/IgM in acute and chronic *Toxoplasma* infection. Biopanda and Onsite POC tests exhibited poor sensitivity for *Toxoplasma*-IgM detection.

*Toxoplasma gondii*, the causative agent of toxoplasmosis, is an obligate intracellular protozoan of worldwide distribution that infects one third of the world’s human population [1]. When *T gondii* infection occurs in immunocompetent patients, it typically follows an asymptomatic and benign clinical course, whereas in immunocompromised hosts, toxoplasmosis can lead to life-threatening manifestations if diagnosed or treated late [2]. Of note, severe presentations of the acute infection in immunocompetent patients have been reported in association with atypical genotypes of the parasite [3, 4]. Primary *T gondii* infection that occurs during pregnancy or within 3 months before conception can result in congenital toxoplasmosis (CT) if not promptly diagnosed and treated [5]. Congenital toxoplasmosis can have devastating consequences for the fetus including stillbirth, neurological sequelae, and severe ocular disease [5]. The risk of CT and the severity of its clinical manifestations are influenced mainly by the gestational age at the time of maternal infection, *T gondii* strain, and antenatal anti-*Toxoplasma* treatment [6]. Maternal *T gondii* infections are usually asymptomatic; approximately half of *T gondii*-infected pregnant women who gave birth to infants with CT did not report any risk factors or any symptoms suggestive of infection [7]. Thus, timely diagnosis of *T gondii* infection during pregnancy relies largely on serological testing throughout gestation in the context of universal prenatal screening programs [8].

Maternal screening programs for CT have been widely adopted in France, Austria, Germany, and other European countries [9–11]. These programs consist of serial serological screening (eg, monthly in France, bimonthly in Austria, and every 2–3 months in Germany) of *Toxoplasma*-seronegative pregnant women coupled with prompt initiation of anti-toxo-plasma treatment upon maternal seroconversion [9–13]. Several observational studies over the past decade concluded that antenatal anti-*Toxoplasma* therapy reduces rates of mother-to-child transmission [9–11, 14] and mitigates the severity of clinical manifestations among congenitally infected infants [9, 11, 15–17]. Early initiation of treatment, within 3–4 weeks from maternal infection, is also critical [14, 16]. However, despite the fact that CT is a preventable and treatable condition, most pregnant women in the United States and worldwide are not routinely screened and treated accordingly for *Toxoplasma* infections [18]. In the United States, CT is usually diagnosed upon appearance of clinical and/or ultrasound findings in the fetus or at birth [5]. Consequently, reported cases of CT in the United States have higher rates of severe clinical manifestations at birth (ie, chorioretinitis, intracranial calcifications, and hydrocephalus) compared with their European counterparts [19–21].

Because approximately 5 to 8 serological tests would be performed in each *Toxoplasma*-seronegative woman, a major factor impeding uptake of monthly maternal screening programs worldwide is high cost of conventional serological testing, which is generally performed at commercial laboratories using automated platforms [22, 23]. Indeed, a decision-analytic economic model in the United States showed that universal monthly maternal screening can be cost saving in the United States, even with an incidence of acute maternal *T gondii* infection as low as 0.2 per 1000 pregnant women, if screening test costs ~$12.00 per test, a figure far below conventional laboratory-based testing cost [24]. Moreover, in Europe, and France in particular, where *Toxoplasma*-seroprevalence in the general population has declined (from 80% in 1960 to 36.7% in 2010) [25], numbers of seronegative pregnant women necessitating screening has increased, thereby endangering further financial viability of screening programs [25, 26]. Hence, alternative diagnostic tools are needed for toxoplasmosis diagnostics.

Point-of-care (POC) rapid tests have improved access to efficient diagnostics across infectious diseases and global health, especially in low-income country settings [27, 28]. Point-of-care technology for antibody detection usually consists of immunochromatographic (ICT) membrane-based assays that can be performed without sophisticated laboratory infrastructure. Previous studies have shown optimal diagnostic performance for the Toxoplasma ICT IgG-IgM (LDBIO Diagnostics, Lyon, France) POC kit when compared with reference-serological methods in France [29, 30] and the United States [31]. However, direct comparisons of performance of various POC kits against reference-standard methods used in the United States, where *T gondii* strains are more diverse, have not been previously performed.

In this study, we sought to evaluate diagnostic accuracy for 3 *Toxoplasma*-IgG-IgM POC kits using a large number of sera from US patients previously tested by reference methods available at Palo Alto Medical Foundation, Toxoplasma Serology Laboratory ([PAMF-TSL] http://www.pamf.org/serology/), the reference laboratory for study and diagnosis of toxoplasmosis in the United States.

## METHODS

### Serological Samples

A total of 310 patient serum samples tested at PAMF-TSL were included: 210 were obtained from the PAMF-TSL biobank, and 100 from the Centers for Disease Control and Prevention (CDC) *Toxoplasma* 1998 Human Serum Panel (CDC-HSP) (Table 1). The PAMF-TSL samples were selected from archived specimens submitted between from February 27, 2013 to June 23, 2017. The CDC-HSP samples were selected from a biobank that contains known positive and negative sera available to researchers and laboratories in the United States. These samples are obtained by the CDC to help evaluate accuracy of commercial *Toxoplasma* antibody test kits (https://www.cdc.gov/dpdx/toxoplasmosis/index.html) and were submitted to investigators in a blinded fashion. All serum samples were previously tested at PAMF-TSL by gold-standard *Toxoplasma*-IgG method (Sabin-Feldman Dye test [DT]), double-sandwich immunoglobulin (Ig)M enzyme-linked immunosorbent assay, and additional confirmatory tests available only at PAMF-TSL.

**Table 1.** Description and Source of Serum Samples

*Toxoplasma*-Infection Status	Source	Total No. Samples	PAMF-TSL Criteria^a^
Acute-*Toxoplasma* infection (n = 85)	PAMF-TSL CDC-HSP	50 35	IgG^+^/IgM^+^ Positive IgG Dye test plus positive IgM-ELISA
Chronic-*Toxoplasma* infection (n = 85)	PAMF-TSL CDC-HSP	50 35	IgG^+/^IgM^−^ Positive IgG Dye test plu*s* negative IgM-ELISA
*Toxoplasma* seronegative (n = 80)	PAMF-TSL CDC-HSP	50 30	IgG^−^/IgM^−^ Negative IgG Dye test plus negative IgM-ELISA
False-positive IgM (n = 60)	PAMF-TSL	60	IgG^−^/IgM^+^ Sera from 33 patients who tested repeatedly positive by IgM-ELISA but failed to show IgG seroconversion (by Dye test) at follow-up testing (≥3 weeks from baseline)
Total samples (n = 310)	PAMF-TSL CDC-HSP	210 100	*Toxoplasma*-IgG^+^: 170/*Toxoplasma*-IgG^−^: 80 *Toxoplasma*-IgM^+^: 85/*Toxoplasma*-IgM^−^: 165

Abbreviations: CDC-HSP, Centers for Disease Control and Prevention *Toxoplasma* 1998 Human Serum Panel; ELISA, enzyme-linked immunosorbent assay; Ig, immunoglobulin; IQR, interquartile range; PAMF-TSL, Palo Alto Medical Foundation, Toxoplasma Serology Laboratory.

^a^PAMF-TSL serological testing include IgG Dye test (positive ≥1:16 dilutions; negative <1:16 dilutions); IgM-ELISA (positive ≥ 2.0 units; indeterminate 1.7–1.9 units; and negative ≤1.6 units). All samples from patients with acute-*Toxoplasma* infection from PAMF-TSL had low IgG avidity (by VIDAS Toxo-IgG Avidity kit; bioMérieux, Marcy-l’Etoile, France); low avidity (cutoff <20%; median, 8.3%; IQR, 4.4%–13.3%; tested) and an acute pattern result when tested with the differential agglutination test (AC/HS test).

The PAMF-TSL samples (n = 210) were divided into 4 different groups: (1) acute-*Toxoplasma* infection (*Toxoplasma*-IgG^+^/IgM^+^ confirmed with additional testing [IgG-avidity, differential agglutination {AC/HS}, *Toxoplasma*-IgA, and *Toxoplasma*-IgE] indicative of a recently acquired infection) (n = 50); (2) chronic-*Toxoplasma* infection (*Toxoplasma*-IgG^+^/IgM^−^) (n = 50); (3) seronegative samples (*Toxoplasma*-IgG^−^/IgM^−^, never infected with *T gondii*) (n = 50); and (4) false-positive (FP) *Toxoplasma*-IgM (*Toxoplasma*-IgG^−^/IgM^+^) (n = 60) [23]. False-positive *Toxoplasma*-IgM serum samples were considered samples that tested repeatedly positive by IgM-enzyme-linked immunosorbent assay ([ELISA] at least in 2 separate test dates separated ≥3 weeks apart) but failed to show IgG seroconversion (when tested by DT) at follow-up testing at PAMF-TSL. In addition to IgM-ELISA and *Toxoplasma*-IgG DT, all of these samples were also tested with additional diagnostic tests, including differential agglutination (AC/HS), *Toxoplasma*-IgA, and *Toxoplasma*-IgE, and were all negative. Moreover, final interpretation of serological results from PAMF-TSL’s consulting physician (J.G.M.) stated that the serologic test results for these samples were compatible with FP *Toxoplasma*-IgM.

Of 100 CDC-HSP samples, 35 were established to have acute *Toxoplasma* infection, 35 to have chronic infection, and 30 uninfected. The POC testing results for CDC-HSP samples were returned to the CDC for data analysis and calculation of IgG and IgM diagnostic sensitivity and specificity for each POC kit.

### Point-of-Care Kits

**Figure 1.**
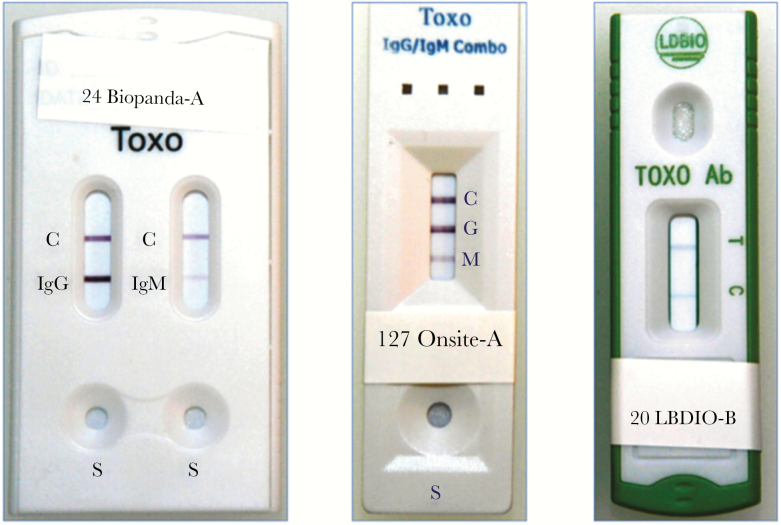
*Toxoplasma* point-of-care (POC) kits for detection of *Toxoplasma* immunoglobulin IgG and IgM antibodies. (Left) Biopanda POC test Toxo IgG/IgM Rapid Test (Biopanda Reagents, Belfast, UK) cassette showing 2 separate strips for *Toxoplasma gondii* IgG and IgM detection, each one with a control band (C) (sample no. 24: positive IgG, positive IgM). (Center) OnSite POC test Toxo IgG/IgM Combo Rapid test (CTK Biotech, San Diego, CA) cassette showing 1 single testing strip with 3 separate testing bands for control (C), *T gondii* IgG (G) and IgM (M) antibodies (from top to bottom) (sample no. 127: positive IgG, positive IgM). (Right) LDBIO POC test Toxoplasma ICT IgG and IgM (LDBIO Diagnostics, Lyon, France) cassette showing 1 testing strip with a testing band for simultaneous detection of *T gondii* IgG/IgM antibodies (T) and a control band (C) (sample no. 20: positive combined IgG/IgM).

Three POC kits were included: (1) Toxo IgG/IgM Rapid Test (Biopanda Reagents, Belfast, UK) (Biopanda POC test); (2) OnSite Toxo IgG/IgM Combo Rapid Test (CTK-Biotech, San Diego, CA) (OnSite POC test); and (3) Toxoplasma ICT IgG-IgM (LDBIO Diagnostics, Lyon, France) (LDBIO POC test). All 3 POC kits are based on a lateral-flow immunochromatography method that uses a nitrocellulose membrane strip containing a test (T) and control (C) band. For the Biopanda POC and OnSite POC tests, their cassette arrangement allows detection and differentiation of *Toxoplasma*-IgG and *Toxoplasma*-IgM antibodies in 2 different testing bands (IgG and IgM). For the LDBIO POC kit, test band (T band) simultaneously detects *Toxoplasma*-IgG and *Toxoplasma*-IgM not allowing their differentiation (Figure 1). The POC testing and interpretation of results were performed (1) following manufacturer’s instructions and (2) in duplicates by 2 independent investigators (L.N.B. and C.A.G.). Final readings were ascertained by these (L.N.B. and C.A.G.) and 2 additional investigators (J.G.M. and D.G.C.-I.). All investigators performing and reading results (L.N.B., C.A.G., J.G.M., and D.G.C.-I.) were blinded to both PAMF-TSL’s and CDC’s results when POC tests were performed.

### Serological Testing at Palo Alto Medical Foundation Toxoplasma Serology Laboratory

*Toxoplasma*-IgG testing was performed using Sabin-Feldman IgG DT, the gold-standard method for *Toxoplasma*-IgG detection in the United States. The DT is a very sensitive and specific test in which live tachyzoites are lysed in the presence of complement and *T gondii*-specific IgG derived from patients’ samples. *Toxoplasma*-IgM testing was performed using a laboratory-developed, double-sandwich IgM-ELISA that uses sonicated *T gondii* antigen prepared from live tachyzoites (www.pamf.org/serology/clinicianguide.html). When indicated (eg, sera with positive *Toxoplasma*-IgM test results), additional tests such as *Toxoplasma*-IgG avidity, differential agglutination (AC/HS), *Toxoplasma*-IgA, and *Toxoplasma*-IgE, were performed by PAMF-TSL [23]. According to PAMF-TSL results, sera from PAMF-TSL biobank and CDC were classified in 4 groups, described above under “Serological Samples”: (1) acute *Toxoplasma* infection; (2) chronic *Toxoplasma* infection; (3) seronegative samples uninfected with *T gondii*; and (4) FP *Toxoplasma*-IgM.

### Statistical Analysis

Sensitivity, specificity, positive predicted values (PPVs), and negative predicted values (NPVs) were calculated against PAMF-TSL IgG and IgM test results, respectively. Diagnostic accuracy was calculated as proportion of correctly classified tests (true positive [TP]+true negative [TN]) among all tests performed (TP+TN+FP+false negative [FN]) [32], as suggested by the Standards for Reporting of Diagnostic Accuracy (STARD) statement [33]. Continuous variables were compared using Student *t* test or Wilcoxon signed-rank test. All tests for significance were 2-sided, and *P* values ≤.05 were considered significant. Statistical analysis was performed using Prism 7.0 software (GraphPad, La Jolla, CA).

## RESULTS

A total of 1860 POC tests (310 samples × 3 POC kits × 2 [duplicate testing of each POC kit]) were performed.

### Diagnostic Accuracy of Point-of-Care Testing for *Toxoplasma*-Immunoglobulin G

Sensitivity calculations for *Toxoplasma*-IgG detection were done for 170 sera (85 from PAMF-TSL biobank and 85 from CDC-HSP). Sensitivity was 100% for all 3 POC kits (95% confidence interval [CI], 97.8%–100%; 170 of 170). Specificity for *Toxoplasma*-IgG detection was comparable among all 3 POC kits: 96.3% (95% CI, 89.5%–98.9%; 77 of 80), 97.5% (95% CI, 91.3%–99.6%; 78 of 80), and 98.8% (95% CI, 93.2%–99.9%; 79 of 80), for Biopanda, OnSite, and LDBIO POC tests, respectively (Tables 2–4). Diagnostic accuracy for IgG detection was 98.8% (247 of 250), 99.2% (248 of 250), and 99.6% (249 of 250) for Biopanda, OnSite, and LDBIO POC tests, respectively.

**Table 2.** Results of Point-of-Care Testing for Detection of *Toxoplasma* IgG and/or IgM Antibodies

POC Kit	*Toxoplasma* Antibodies	IgG^+^ (Positives/Reference Testing at PAMF-TSL) (%)	IgG^−^/IgM^−^ (Positives/Reference Testing at PAMF-TSL) (%)	*Toxoplasma* Antibodies	IgM^+a^ (Positives/Reference Testing at PAMF-TSL) (%)	IgM^−a^ (Positives/Reference Testing at PAMF-TSL) (%)
Biopanda	IgG	170 of 170 (100)	3 of 80 (3.8)	IgM	51 of 82^b^ (62.2)	19 of 165 (11.5)
OnSite	IgG	170 of 170 (100)	2 of 80 (2.5)	IgM	23 of 82^b^ (28)	4 of 165 (2.4)
LDBIO^c^	IgG/IgM Combined	170 of 170 (100)	1 of 80 (1.3)	IgG/IgM Combined	82 of 82^b^ (100)	NA

Abbreviations: CDC, Centers for Disease Control and Prevention; CDC-HSP, CDC *Toxoplasma* 1998 Human Serum Panel; Ig, immunoglobulin; NA, not applicable; PAMF-TSL, Palo Alto Medical Foundation, *Toxoplasma* Serology Laboratory; POC, point of care.

^a^All IgM positive sera were also IgG positive by reference testing. The IgM negative sera were either IgG^+^IgM^−^ sera (n = 85) or IgG^−^IgM^−^ sera (n = 80).

^b^Three of 85 samples (from the CDC-HSP 1998) were excluded from the calculation of analytical sensitivity for IgM that were provided to us by the CDC report, because PAMF-TSL IgM-ELISA results were <2.0 units (cutoff for positive).

^c^LDBIO POC test detects in combination *Toxoplasma* IgG/IgM.

**Table 3.** Results of Point-of-Care Testing for Detection of *Toxoplasma* IgG/IgM per *Toxoplasma* Infection Status

POC Kit	*Toxoplasma* Antibodies	POC Result	*Toxoplasma* Infection Status		
			Acute Infection (IgG^+^/IgM^+^) n = 85^a^ (%)	Chronic Infection (IgG^+^/IgM^−^) n = 85 (%)	Seronegative (IgG^−^/IgM^−^) n = 80 (%)
Biopanda	IgG	Positive	85 (100)	85 (100)	3 (3.8)
		Negative	0 (0)	0 (0)	77 (96.3)
	IgM	Positive	51 (62.2)	16 (18.9)	3 (3.8)
		Negative	31 (37.8)	69 (81.1)	77 (96.2)
OnSite	IgG	Positive	85 (100)	85 (100)	2 (2.5)
		Negative	0 (0)	0 (0)	78 (97.5)
	IgM	Positive	23 (28.0)	3 (3.5)	1 (1.3)
		Negative	59 (72.0)	82 (96.5)	79 (98.7)
LDBIO	IgG/IgM Combined	Positive	85 (100)	85 (100)	1 (1.3)
		Negative	0 (0)	0 (0)	79 (98.7)

Abbreviations: CDC, Centers for Disease Control and Prevention; CDC-HSP, CDC *Toxoplasma* 1998 Human Serum Panel; ELISA, enzyme-linked immunosorbent assay; IgG, immunoglobulin; PAMF-TSL, Palo Alto Medical Foundation, *Toxoplasma* Serology Laboratory; POC, point of care.

^a^ Three of 85 samples (from the CDC-HSP 1998) were excluded from the calculation of analytical sensitivity for IgM that were provided to us by the CDC report, because PAMF-TSL IgM-ELISA results were <2.0 units (cutoff for positive).

**Table 4.** Diagnostic Accuracy of Three POC Kits for *Toxoplasma* IgG and IgM Detection

POC Kit	*Toxoplasma* Antibodies	Sensitivity (%) (95% CI)	Specificity (%) (95% CI)	NPV^b^ (%) (95% CI)	PPV^b^ (%) (95% CI)
Biopanda	IgG	100 (97.8–100)	96.3 (89.5–98.9)	100 (95.3–100)	98.3 (95.0–99.5)
	IgM	62.2 (51.4–71.9)	88.5 (82.7–92.5)	82.5 (76.2–87.4)	72.9 (61.5–81.9)
OnSite	IgG	100 (97.8–100)	97.5 (91.3–99.6)	100 (95.3–100)	98.8 (95.8–99.8)
	IgM	28.0 (19.5–38.6)	97.6 (93.9–99.1)	73.2 (66.9–78.6)	85.2 (67.5–94.1)
LDBIO^a^	IgG/IgM Combined	100 (97.8–100)	98.8 (93.2–99.9)	100 (95.4–100)	99.4 (96.8–99.9)

Abbreviations: CI, confidence interval; Ig, immunoglobulin; POC, point of care; NPV, negative predictive value; PPV, positive predictive value.

^a^As the LDBIO-POC test reports *Toxoplasma* IgG and IgM antibodies combined in a single testing band (T band), specificity for LDBIO-POC test was calculated over 80 seronegative sera (*Toxoplasma*-IgG^−^/IgM^−^)

^b^The above PPV and NPV for the 3 POC tests are representative of the performance of these tests in a group of samples in which 33% were *Toxoplasma* IgM^+^ and 68% were *Toxoplasma* IgG^+^. In pragmatic clinical settings, the prevalence of *Toxoplasma* IgM^+^ individuals is significantly lower (usually <1% [<10 of 1000]).

Detailed serological results for *Toxoplasma*-IgG and *Toxoplasma*-IgM titers were available only for sera from PAMF-TSL biobank. For the 100 *Toxoplasma*-IgG^+^ patients from PAMF-TSL biobank, median IgG DT titer was 1:512 (range, 1:32 to 1:32000). The IgG titers from patients with acute *Toxoplasma* infection were significantly higher compared with patients with chronic *Toxoplasma* infection (median 8000 [interquartile range {IQR}, 2048–8000] vs 192 [IQR, 64–512], respectively; *P* < .0001). Of note, all 3 POC kits showed optimal sensitivity for IgG detection across all tiers of IgG DT titers. Diagnostic performance for all POC kits according to infection status is shown in Table 3.

### Diagnostic Accuracy of Point-of-Care Testing for *Toxoplasma*-Immunoglobulin M

Sensitivity calculations for detection of *Toxoplasma*-IgM was performed for 82 (instead of 85) *Toxoplasma*-IgM-positive serum samples because 3 of 35 positive *Toxoplasma*-IgM samples from the CDC-HSP serum panel were dilutions of 3 true *Toxoplasma*-IgM-positives specimens, and their corresponding PAMF-TSL IgM-ELISA results were <2.0 (0.5, 0.8, and 1.6 units). Therefore, these 3 samples were excluded from sensitivity calculation in results reported to us by the CDC (Tables 2 and 3).

Sensitivity for *Toxoplasma*-IgM varied significantly across POC kits: Biopanda POC test 62.2% (95% CI, 51.4%–71.9%; 51 of 82), OnSite-POC test 28% (95% CI, 19.5%–38.6%; 23 of 82), and LDBIO-POC test for combined IgG/IgM detection 100% (95% CI, 95.5%–100%; 82 of 82) (*P* < .0001 for all comparisons). Specificity for *Toxoplasma*-IgM was significantly lower for Biopanda compared with OnSite and LDBIO POC tests: 88.5% (95% CI, 82.7%–92.5%; 146 of 165), 97.6% (95% CI, 93.9–99.1%; 161 of 165), and 98.8% (95% CI, 93.2%–99.9%; 79 of 80), respectively (*P* = .0012 for Biopanda vs OnSite POC tests; *P* = .005 for Biopanda vs LDBIO POC tests) (Table 4). Diagnostic accuracy for IgM detection was significantly lower for Biopanda and OnSite POC tests in comparison with LDBIO POC test: 79.8% (197 of 247), 74.5% (184 of 247), and 99.4% (161 of 162), respectively (*P* < .0001 for both Biopanda vs LDBIO POC test and OnSite vs LDBIO POC test).

### Cross-Reactivity of Point-of-Care Kits With Nonspecific Immunoglobulin M

All 3 POC kits showed negative results for IgM (0 of 60) when tested against FP *Toxoplasma*-IgM samples (nonspecific IgM) (Table 5). In 3 of 60 (5%) samples, the Biopanda POC kit tested falsely positive for IgG. Of note, IgM-ELISA titers from patients with nonspecific IgM were significantly lower compared with patients with true acute *Toxoplasma* infection (median 3.3 [IQR, 2.7–4.4] vs 6.8 [IQR, 5.0–8.4], respectively; *P* < .0001).

**Table 5.** Results of POC Testing Against Selected Sera With False-Positive IgM Results for *Toxoplasma* IgM

POC Kit	*Toxoplasma* Antibodies	Positive/Total (%)
Biopanda	IgG	3 of 60 (5%)
	IgM	0 of 60 (0%)
OnSite	IgG	0 of 60 (0%)
	IgM	0 of 60 (0%)
LDBIO	IgG/IgM combined	0 of 60 (0%)

Abbreviations: Ig, immunoglobulin; POC, point-of-care.

### Interobserver and Intertest Agreement

There were instances when Biopanda and OnSite POC cassettes displayed poorly visualized testing bands (ie, very faint colored bands-ambiguous bands) (Supplementary Material, Figure S1). Ambiguous bands were seen in 44 of 620 (7.1%) of Biopanda POC tests (5 for IgG and 39 for IgM bands); 13 of 39 (33%) of ambiguous IgM bands were seen in both POC duplicate tests for a given sample. For OnSite, ambiguous bands were seen in 28 of 620 (4.5%) POC tests (2 for IgG and 26 for IgM bands); 19 of 26 (73%) of ambiguous IgM bands were seen in both POC duplicate tests. However, because Biopanda and OnSite POC tests manufacturer’s instructions indicate that any shade of color on testing bands should be interpreted as positive, all ambiguous-bands for these 2 POC tests were re-evaluated by 2 other investigators (J.G.M., D.G.C.-I.) blinded also to both PAMF-TSL results, and their corresponding duplicate POC test and results were classified accordingly. There were no ambiguous bands for LDBIO POC tests. Lastly, per manufacturer instructions, all 1860 POC test results were considered valid because all of them exhibited the presence of the control (C) band.

## DISCUSSION

We report the diagnostic performance of 3 POC tests for detection of *Toxoplasma* IgG and IgM antibodies using a large number of sera from US patients tested by PAMF-TSL reference gold-standard IgG and IgM tests. There are 2 major conclusions from our study. First, all POC tests (Biopanda, OnSite, and LDBIO) demonstrated optimal analytical sensitivity and specificity for *Toxoplasma*-IgG testing irrespectively of *Toxoplasma*-infection status (acute vs chronic infection) and IgG titers. Second, except for LDBIO POC test that detects IgG-IgM in one testing band and therefore showed 100% diagnostic sensitivity for acute *Toxoplasma* infection, Biopanda and OnSite POC tests showed poor diagnostic sensitivity for *Toxoplasma*-IgM detection (62.2% and 28%, respectively). Notably, no POC tests showed cross-reactivity with sera from patients with FP *Toxoplasma*-IgM results. Hence, our results support use of the LDBIO-POC platform as a simple, low-cost, and rapid diagnostic tool capable of efficiently detecting *Toxoplasma*-IgG and/or *Toxoplasma*-IgM antibodies. Our study also supports the LDBIO-POC test as an initial serological screening test in programs for universal *Toxoplasma* prenatal testing.

The underperforming diagnostic accuracy for *Toxoplasma*-IgM detection noted for the 2 POC tests that were designed to detect IgG and IgM separately deserves further discussion. There are some technical differences between these 3 POC tests. The Biopanda and OnSite POC tests use recombinant *T gondii* antigens for binding to specific anti-*Toxoplasma* antibodies (IgG and IgM, respectively), whereas the LDBIO POC test uses as antigen whole-cell lysates of tachyzoites from the *T gondii* RH Sabin Type I strain. Additional mechanistic differences on lateral flow assays design may also account for the higher diagnostic performance observed with the LDBIO POC kit (see Supplementary Material). Biopanda and OnSite offer in their POC cassettes detection of *Toxoplasma*-IgM antibodies in a distinct testing band (M band, Figure 1). False-negative results for IgM may lead to delay confirmatory testing, missing the opportunity for timely initiation of anti-*Toxoplasma* therapy in seroconverting pregnant women. In addition, the Biopanda and the OnSite POC tests exhibited the presence of ambiguous bands, defined as poorly visualized testing bands with very faint color, which were seen in 7.1% (44 of 620) and 4.5% (28 of 620) of the tests performed by Biopanda and OnSite, respectively. The above, added to an excessive number of FP results (11.5% for Biopanda IgM), represents a significant drawback that, in clinical practice, may lead to confusion and unnecessary confirmatory testing at expenses of increasing testing cost and patient’s anxiety. The failure of the Biopanda (62.2% sensitivity) and OnSite POC tests (28% sensitivity) to optimally detect *Toxoplasma* IgM represents a serious handicap for the implementation of such POC tests in universal screening programs during pregnancy.

The LDBIO Toxoplasma POC kit showed robust performance in our study, with 100% sensitivity for the combined detection of IgG and IgM detection and low rate of FP IgG/IgM POC test results (1.3%, 1 of 80 in IgG/IgM seronegative sera). Of note, the LDBIO reports *Toxoplasma*-IgG and *Toxoplasma*-IgM antibodies combined in a single testing band (T band); therefore, independent calculation of IgM sensitivity and specificity for this POC test, without taking into account the presence of IgG, was not possible (Tables 2 to 3). The diagnostic accuracy for LDBIO has been documented in prior studies using various reference standard methods for *Toxoplasma*-IgG and IgM detection [29–31]. To date, at least ~2000 sera and whole blood samples from France, United States, and Morocco have been tested (including the 310 sera from this study). Chapey et al [29] evaluated the LDBIO POC test in 400 sera from France and demonstrated diagnostic sensitivity and specificity of 97% and 96%, respectively, for the combined detection of *Toxoplasma* IgG-IgM when compared with the fully automated chemiluminescence Architect test (Abbott North, Chicago, IL). Our team, led by McLeod et al demonstrated 100% sensitivity and specificity for the LDBIO POC test when tested against 180 sera from United States patients, including seropositive individuals with diverse *T gondii* serotypes previously tested at PAMF-TSL [31]. Mahinc et al tested 1002 sera from France and reported 100% analytical sensitivity in comparison with *Toxoplasma*-IgG II Western blot analysis and Platelia Toxo-IgM (Bio-Rad) for IgG and IgM detection, respectively. In Mahinc et al’s [30] study, the LDBIO POC kit showed reliable performance even at low IgG titers, outperforming the Architect IgG test in its gray zone of reporting. More recently, the McLeod-Lykins team tested LDBIO POC test’s suitability using fingerstick whole blood in 244 whole blood samples from 205 consenting individuals from the National Chicago-Based, Congenital Toxoplasmosis Collaborative Study (n = 208 samples) and Morocco (n = 39 samples), including 101 seropositive sera, demonstrating 100% sensitivity and specificity when compared with automated commercial laboratory testing for IgG and IgM or PAMF-TSL testing [34]. In light of accumulated evidence for LDBIO POC test’s accuracy in comparison with reference-standard methods in the United States and France, the LDBIO POC kit can be deemed as an optimal low-cost technology for population-based serological screening and/or universal monthly prenatal screening. This POC test has been shown to perform well independently of implicated *T gondii* strain and across a wide range of *Toxoplasma*-IgG antibody titers [31]. If cost of LDBIO POC kit can be maintained at <$5.00, this assay may become an attractive option for first-line testing across maternal screening programs.

Our study has some limitations. Despite large sample size and inclusion of sera from acute and chronic *Toxoplasma* infection and FP IgM results, we did not include sera from patients with early *T gondii* infection (early seroconversion) to assess for IgM diagnostic accuracy in the absence of *Toxoplasma* IgG. These sera are scarce given lack of routine prenatal screening for *Toxoplasma* infection in the United States, impeding access to patients with documented seroconversion at early stages of infection. Therefore, in our study, IgM titers from patients with acute *Toxoplasma* infection were remarkably high (IgM-ELISA titers; median 6.8 [IQR, 5.0–8.4]; cutoff ≥2.0 units). As a result, POC analytical performance for IgM detection at lower IgM titers remains unknown, and we encourage investigators to address this issue in future prospective studies. Reassuringly, Mahinc et al [30] tested sera from 17 individuals with acute seroconversion that were only IgM positive and IgG negative and provided proof of concept that the LDBIO POC test can detect early *Toxoplasma* IgM (16 of 17 positive).

Because our study evaluated the diagnostic accuracy of POC kits compared with reference laboratory methods for *Toxoplasma* serology testing, the estimated PPVs and NPVs were representative of a group of tested samples with 68% IgG and 33% IgM seropositivity. Positive predicted values and NPVs vary according to the prevalence of the disease or condition in a determined population, and thus the PPVs and NPVs for a pragmatic cohorts with different IgG and IgM seropositivity rates would be very different; eg, for a population with 1% IgM seropositivity, a POC test with sensitivity and specificity for IgM detection of 62.2% and 88.5%, respectively, would have a PPV of only 5.2%, and a NPV of 99.6% and a POC test with 28% and 97.6% sensitivity and specificity, respectively, would have a PPV of 10.5% and NPV of 99.26%.

Our study was a proof-of-concept study and the first to directly compare 3 commercial POC tests for *Toxoplasma* diagnostics. As such, our results have notable implications for obstetricians, pediatricians, neonatologists, infectious diseases specialists, and others including policy makers in public health. As a neglected disease, CT represents a significant burden to healthcare systems around the world, with 190000 infants per annum affected worldwide carrying 1.2 million disability-adjusted life years [35–37]. Integration of POC platforms into *Toxoplasma* diagnostics holds enormous promise given its critical elements such as rapid turnaround time, simplicity, low cost, and completion of testing during same clinical encounter [38]. However, careful evaluation of POC test accuracy and diagnostic performance is a pivotal step before any consideration for clinical use. The World Health Organization has proposed the “ASSURED” criteria for the ideal POC assay: Affordable, Sensitive, Specific, User-friendly, Rapid/robust, Equipment-free and Deliverable to users [28]. In this frame, our study represents the initial stage to move forward POC diagnostics in toxoplasmosis. Given their low complexity and minimal risk for incorrect results, POC kits with robust analytical performance such as the LDBIO POC test, which has also been shown to have excellent diagnostic accuracy when tested with whole blood collected directly from a fingerstick (via a capillary tube) [34], can receive Clinical Laboratory Improvement Amendments waivers, enabling them to be used in POC settings and thereby contribute to mitigate costs associated with serological testing in maternal screening programs [24]. Despite enthusiasm, several questions regarding the application of POC diagnostics in clinical grounds remain to be addressed, and future studies are needed to prospectively evaluate effectiveness of POC test-based prenatal screening strategies, including cost effectiveness in individual country settings, impact on clinical outcomes, and their integration to clinical workflows in perinatal care [39]. Work is in progress for the evaluation of POC test-based prenatal screening integration in the clinical workflow in prenatal care (Thrasher Research Fund award no. 13798).

Positive POC test results indicating acquisition of an acute *Toxoplasma* infection during gestation (eg, seroconversion from IgG^−^/IgM^−^ serostatus to an IgM^+^ or IgG^+^ or combined IgG^+^/IgM^+^ status between 2 consecutive screenings) would always require confirmatory testing with conventional laboratory-based methods to confirm the diagnosis and guide clinical management. Moreover, positive POC test results at the first prenatal care visit (either IgG^+^/IgM^−^ or positive for the combined IgG/IgM detection) also require confirmatory testing, to confirm that these reflect chronic infection, acquired before gestation. Pregnant women found to be chronically infected early in gestation would not need additional monthly screening for the rest of their pregnancy because they are not at risk of transmitting the infection to their fetus, unless severely immunocompromised and not on prophylactic therapy. Despite the need for confirmatory testing for positive results, POC test-based prenatal screening programs are still cost saving because they significantly limit the number of women who will need such confirmatory testing (even in settings with seropositivity rates among pregnant women as high as 50%, a POC test-based screening program can cut the need for laboratory-based screening by half).

## CONCLUSIONS

In conclusion, we demonstrated that all 3 POC tests (Biopanda, OnSite, and LDBIO POC tests) showed optimal diagnostic sensitivity and specificity for *Toxoplasma*-IgG detection across different *Toxoplasma*-infection states. However, the LDBIO POC test showed 100% sensitivity for the combined detection of IgG/IgM in acute and chronic *Toxoplasma* infection, whereas Biopanda and OnSite POC tests showed poor diagnostic sensitivity for *Toxoplasma* IgM. An accurate, low-cost, and reliable POC diagnostic test for *Toxoplasma* infection can benefit patients and mitigate the cost of serological testing in universal prenatal screening programs for *Toxoplasma* infections.

## Supplementary Data

Supplementary materials are available at *Open Forum Infectious Diseases* online. Consisting of data provided by the authors to benefit the reader, the posted materials are not copyedited and are the sole responsibility of the authors, so questions or comments should be addressed to the corresponding author.

ofy215_suppl_supplementary_material

## Acknowledgments

***Financial support.*** This work was funded by the E.W, AI Thrasher Foundation (number 13796; to Y. M., D. G. C.-I., J. G. M., and R. M.) and the Mann Cornwell, Rooney, Morel, Taub, and Engel families.

***Potential conflicts of interest.*** R. M. and J. G. M. performed a literature review for Sanofi-Pasteur during the time this manuscript was prepared; no compensation was received for this review. All authors have submitted the ICMJE Form for Disclosure of Potential Conflicts of Interest. Conflicts that the editors consider relevant to the content of the manuscript have been disclosed.
