# Supplementary material for: Evaluation of Three Point-of-Care Tests for Detection of Toxoplasma Immunoglobulin IgG and IgM in the United States: Proof of Concept and Challenges
Source: Open Forum Infect Dis. 2018 Oct 29;5(10):ofy215. doi: 10.1093/ofid/ofy215 (PMC6204989; doi:10.1093/ofid/ofy215)
Supplement: ofy215_suppl_supplementary_material [file ofy215_suppl_supplementary_material.docx]

**SUPPLEMENTARY MATERIAL**

**Materials and Methods**

POC-tests used in this study:

1. **Biopanda-POC-test,** *Toxo IgG/IgM Rapid-test* (Biopanda Reagents, Belfast, UK): This POC-kit is comprised of a test cassette with 2 nitrocellulose membrane strips for testing: one for IgG and one for IgM. Each test cassette has a control (C) band, a testing (T) band and a corresponding sample well. Mouse anti-human IgG and IgM are coated in the testing line (for IgG and IgM respectively). This POC-kit use recombinant *T. gondii* antigens for binding to specific anti-*toxoplasma* antibodies (IgG and/or IgM) present in the patient’s sample, forming antibody-antigen (Ab-Ag) complexes. These complexes travel through the cassette membrane, via chromatographic migration, and then are captured by mouse anti-human IgG and IgM antibodies coated in the T band. Testing is performed by depositing a sample of serum in each sample well (20 μl, ~1 drop via capillary tube) and then an eluent solution (2 drops of buffer). The reading must be done between 15 and 20 minutes.
2. **Onsite-POC-test**, *Onsite Toxo IgG/IgM Combo Rapid-test* (CTK-Biotech, San Diego, USA): The test strip in this cassette device consists of: 1) a burgundy colored conjugate pad containing a recombinant *T. gondii* antigen conjugated with colloidal gold (Toxo-conjugates) plus a control antibody conjugated with colloidal gold and 2) a nitrocellulose membrane strip containing two test lines (G and M lines) and a control line (C line). The G line is pre-coated with antihuman IgG for detection of IgG anti-*T. gondii*. The M line is pre-coated with mouse anti-human IgM for detection of IgM anti-*T. gondii*. The C line is pre-coated with a control antibody. Testing is performed by depositing a sample of serum in each sample well (10 μl) (10 μl capillary tubes provided with the POC-kit) and then an eluent solution (2 drops of sample diluent). The reading must be done at 10 minutes. Negative results should be confirmed at the end of 15 minutes only.
3. **LDBIO-POC-test,** *Toxoplasma-ICT IgG-IgM-bk* (LDBIO Diagnostics, Lyon, France) consists of a nitrocellulose strip on which are spread two reactive bands: the antigens (*Toxoplasma gondii*) of the “test” band (T band) and the rabbit gamma globulins of the “control” band (C band). LDBIO-POC-test uses latex particles as conjugate carriers of anti-*toxoplasma* antibodies and their capture at the testing line is mediated by coated *T. gondii* antigens derived from whole-cell lysates of tachyzoites (*T. gondii* RH Sabin Type I strain) (personal communication, LDBIO-Diagnostics to DCI and JGM). The fiberglass support (conjugate pad), which is impregnated of black latex particles, coupled with *Toxoplasma* antigens (“test” latex = T latex) and blue latex particles coupled with goat anti-rabbit IgG (“control” latex = C latex) contains the sample well. The test is run by successively dispensing the sample (15 μl) (serum, plasma or whole blood) and an eluting solution (called the eluent) (4 drops) in the “sample well” of the cassette. Adding the eluent starts the concomitant migration (chromatography) of the serum and the latex particles. This migration is completed in 20-30 minutes. If anti-*Toxoplasma* antibodies (IgG and/or IgM) are present in the sample, a complex is formed between the T latex and the patient’s antibodies which is then captured by the T band and it results in the appearance of a black line. The test is positive when 2 lines (T and C) appear in their corresponding areas. The reading must be done between 20 and 30 minutes.

**Interpretation of POC-results:**

The investigators performing the reading of the POC-test results had followed the specific *“Interpretation of Results”* instructions provided by the manufacturing companies within their product inserts, as it would have been done in a pragmatic clinical setting.

Results were interpreted at their corresponding time of reading following POC-test’s manufacturers instructions. POC-tests results without the presence of a control (C) band are interpreted as invalid per POC-test’s manufacturers instructions. Reading and interpretation of POC-results were performed in blinded fashion and independently of other POC-tests for the corresponding serum sample. All POC-tests were performed in duplicates (A&B) and POC-test’s result interpretation was performed independently for each duplicate POC-test. Results were recorded in paper-spreadsheet and entered in real-time into an Excel spreadsheet file. Initial interpretation of POC (A and B duplicate) was performed at the testing site following POC-testing by 2 investigators (LNB and CAG) and pictures were taken for each POC-kit result using a digital camera on projector (AVerVision, Model spb370, Aver, Freemont, CA, USA, [www.averusa.com](http://www.averusa.com)). Pictures were saved in JPEG mode (5 megapixels), and stored in secure mode in a password protected USB drive. A second POC-tests reading was performed by an additional investigator (DCI) using photographic material. These results were recorded in a separate excel spreadsheet. If agreement between A & B and between first and second reader was accomplished, results were considered finalized. In case of any disputes or disagreements (e.g., ambiguous bands: very faint color bands), pictures were sent to a third investigator for arbitration (JGM). Interpretation by this investigator was considered as the final reading. Of note, all investigators performing and reading the results (LNG, CAG, JGM, and DCI) were blinded to both PAMF-TSL’s and CDC’s results at the time the POC tests were performed and read.

Once interpretation-results for the 100 CDC-HSP samples were finalized, these results were submitted back to CDC for diagnostic accuracy analysis, separately for each POC-test. For the Biopanda- and the Onsite POC-tests, the CDC provided us: a) the sensitivity and specificity and 95% confidence intervals thereof, separately for each POC-test IgG and POC-test IgM; b) the number of true positive POC-test IgG results (and POC-test-IgM-results respectively) ; c) the total number of positive IgG (and IgM respectively) samples in the pool of the tested 100 CDC-HSP samples; d) the total number of negative IgG (and IgM respectively) samples in the pool of the tested 100 CDC-HSP samples and e) the number of false positive POC-test IgG results (and POC-test IgM-results respectively).

For the LDBIO-POC-test, the diagnostic accuracy was provided to us for the combined detection of IgG and IgM. Specifically, CDC provided us: a) the LDBIO sensitivity and specificity and 95% confidence intervals thereof, for the combined LDBIO-POC-test combined IgG/IgM detection; b) the number of true positive LDBIO-POC-test combined IgG/IgM test results and c) the number of false positive LDBIO-POC-test combined IgG/IgM results.

After receiving the interpretation of the results for the 3 POC-tests from the 100 CDC-HSP, we proceeded with the calculation of the overall sensitivity, specificity, PPV, NPV and diagnostic accuracy for our study, for each POC-test separately, combining the results from all 310 tested samples (210 from PAMF-TSL plus 100 from CDC-HSP). In this Supplement we also report separately the diagnostic accuracy results from the PAMF-TSL samples and from the CDC-HSP.

Moreover, after finalizing the diagnostic accuracy analyses for all 310 samples; an investigator with access to PAMF-TSL’s *Toxoplasma*-testing results (CP), who was not involved in performing POC-testing, provided PAMF-TSL test results for further sensitivity data analyses.

**Serum samples with false-positive *Toxoplasma*-IgM (nonspecific IgM) (n=60)**

These sera were selected from the PAMF-TSL’s biobank after searching for serum samples that tested repeatedly positive by IgM-ELISA (at least in 2 separate test dates, separated ≥ 3 weeks apart) but failed to show IgG seroconversion at follow-up testing. In addition to IgM-ELISA, PAMF-TSL serological testing included *Toxoplasma*-IgG dye test, differential agglutination (AC/HS), *Toxoplasma* -IgA, and *Toxoplasma*-IgE [1]. To be included in this group, interpretation of serological results from PAMF-TSL’s consulting physician (JGM) must have stated that serological results were compatible with false-positive IgM. Thus, 60 serum samples from 33 unique patients were identified and used for this analysis.

**Supplementary Results**

**Table S1. LDBIO-POC-test against PAMF-TSL sera (n=150)**

| **POC-kit** | **Result** | **Reference Standard testing at PAMF-TSL** | | |
| --- | --- | --- | --- | --- |
|  |  | **IgG+ (n=100)** | **IgM + (n=50)* (Included in IgG+)** | **IgG —/IgM — (n=50)** |
| ***Toxoplasma* ICT IgG-IgM LDBIO** | **Positive** | 100 | 50 | 1 |
|  | **Negative** | 0 | 0 | 49 |
|  | **Total** | 100 | 50 | 150 |
| *All 50 positive were also *Toxoplasma*-IgG positive specimens | | | |  |

**Table S2. LDBIO-POC-test against CDC-HSP (n=100)**

| **POC-kit** | **Result** | **Reference Standard testing at PAMF-TSL** | | |
| --- | --- | --- | --- | --- |
|  |  | **IgG+ (n=70)** | **IgM + (n=35)* (Included in IgG+)** | **IgG —/IgM — (n=30)** |
| ***Toxoplasma* ICT IgG-IgM LDBIO** | **Positive** | 70 | 32 | 0 |
|  | **Negative** | 0 | 0 | 30 |
|  | **Total** | 70 | 32 | 30 |
| All 35 IgM positive were also *Toxoplasma* IgG positive specimens.  ^a^3/85 samples (from the CDC-HSP 1998) were excluded from the calculation of analytical sensitivity for IgM that were provided to us by the CDC-report, because PAMF-TSL IgM-ELISA results were < 2.0 units (cutoff for positive). | | | | |

**Table S3. Biopanda-POC-test against PAMF-TSL sera (n=150)**

| **POC-kit** | **Test band** | **Result** | **Reference Standard testing at PAMF-TSL** | | | |
| --- | --- | --- | --- | --- | --- | --- |
|  |  |  | **IgG + (n=100)** | **IgG —/IgM — (n=50)** | **IgM + (n=50)*** | **IgM — n=100) (Including 50 IgG +)** |
| *Toxo IgG/IgM Rapid-test*  **Biopanda** | **IgG** | **Positive** | 100 | 2 | NA | NA |
|  |  | **Negative** | 0 | 48 | NA | NA |
|  | **IgM** | **Positive** | NA | 2 | 31 | 15** |
|  |  | **Negative** | NA | 48 | 19 | 85 |
|  |  | **Total** | 100 | 50 | 50 | 100 |
| *All 50 IgM positive were also *Toxoplasma*-IgG positive specimens. NA, not applicable.  **13/15 False-positive Biopanda IgM occurred in the group of chronic *toxoplasma* infection (IgG+/ IgM-) | | | | | | |

**Table S4. Biopanda-POC-test against CDC-HSP (n=100)**

| **POC-kit** | **Test band** | **Result** | **Reference Standard testing at PAMF-TSL** | | | |
| --- | --- | --- | --- | --- | --- | --- |
|  |  |  | **IgG +**  **(n=70)** | **IgG —/IgM — (n=30)** | **IgM + (n=35)^a^** | **IgM — (n=65)**  **(Including 30 IgG — and 35 IgG +)** |
| *Toxo IgG/IgM Rapid-test*  **Biopanda** | **IgG** | **Positive** | 70 | 1 | NA | NA |
|  |  | **Negative** | 0 | 29 | NA | NA |
|  | **IgM** | **Positive** | NA | NA | 20 | 4* |
|  |  | **Negative** | NA | NA | 12 | 61 |
|  |  | **Total** | 100 | 30 | 32 | 65 |

All 35 IgM-positive were also *Toxoplasma-*IgG positive specimens.

^a^3/85 samples (from the CDC-HSP 1998) were excluded from the calculation of analytical sensitivity for IgM that were provided to us by the CDC-report, because PAMF-TSL IgM-ELISA results were < 2.0 units (cutoff for positive).

*The 4 IgM false-positive results occurred 3/4 (75%) in IgG+/IgM- sera and in 1/4 (25%) in IgG-/IgM- serum sample

**Table S5. Onsite-POC-test against PAMF-TSL sera (n=150)**

| **POC-kit** | **Test band** | **Result** | **Reference Standard testing at PAMF-TSL** | | | |
| --- | --- | --- | --- | --- | --- | --- |
|  |  |  | **IgG + (n=100)** | **IgG —/IgM — (n=50)** | **IgM + (n=50)*** | **IgM — n=100) (including 50 IgG +)** |
| *Toxo IgG/IgM Combo Rapid-test*  **Onsite** | **IgG** | **Positive** | 100 | 2 | NA | NA |
|  |  | **Negative** | 0 | 48 | NA | NA |
|  | **IgM** | **Positive** | NA | 1 | 17 | 3** |
|  |  | **Negative** | NA | 49 | 33 | 97 |
|  |  | **Total** | 100 | 50 | 50 | 100 |
| *All 50 specimens IgM positive were also *Toxoplasma*-IgG positive specimens. NA, not applicable. **2/3 False-positive Onsite IgM occurred in the group of chronic *toxoplasma* infection (IgG+/ IgM-) | | | | | | |

**Table S6. Onsite-POC-test against CDC-HSP (n=100)**

| **POC-kit** | **Test band** | **Result** | **Reference Standard testing at PAMF-TSL** | | | |
| --- | --- | --- | --- | --- | --- | --- |
|  |  |  | **IgG + (n=70)** | **IgG —/IgM — (n=30)** | **IgM + (n=35)^a^** | **IgM — (n=65)**  **(Including 30 IgG — and 35 IgG +)** |
| *Toxo IgG/IgM Combo Rapid-test*  **Onsite** | **IgG** | **Positive** | 70 | 0 | NA | NA |
|  |  | **Negative** | 0 | 30 | NA | NA |
|  | **IgM** | **Positive** | NA | NA | 6 | 1* |
|  |  | **Negative** | NA | NA | 26 | 64 |
|  |  | **Total** | 100 | 30 | 32 | 65 |

All 35 specimens IgM-positive were also *Toxoplasma-*IgG positive specimens.

^a^3/85 samples (from the CDC-HSP 1998) were excluded from the calculation of analytical sensitivity for IgM that were provided to us by the CDC-report, because PAMF-TSL IgM-ELISA results were < 2.0 units (cutoff for positive).

*The specimen that tested IgM false-positive with Onsite was IgG+/IgM- specimen

**Figure S1. Examples of “Ambiguous bands” (poorly-visualized testing bands due to very faint color), presented with Biopanda- and Onsite-POC-tests (red arrows).** Ambiguous bands were seen in 44/620 (7.1%) of *Biopanda* POC-tests (5 for IgG and 39 for IgM bands) with 13/39 (33%) of ambiguous IgM bands seen in both POC-duplicate tests for a given sample (A &B). For the *Onsite-POC-test,* ambiguous bands were seen in 28/620 (4.5%) of POC-tests (2 for IgG and 26 for IgM bands) with 19/26 (73%) of ambiguous IgM bands seen in both POC-duplicate tests (A&B). For the final reading-interpretation all results were categorized either as positives or as negatives, according to the manufacturers’ instructions for Biopanda- and Onsite-POC-test.


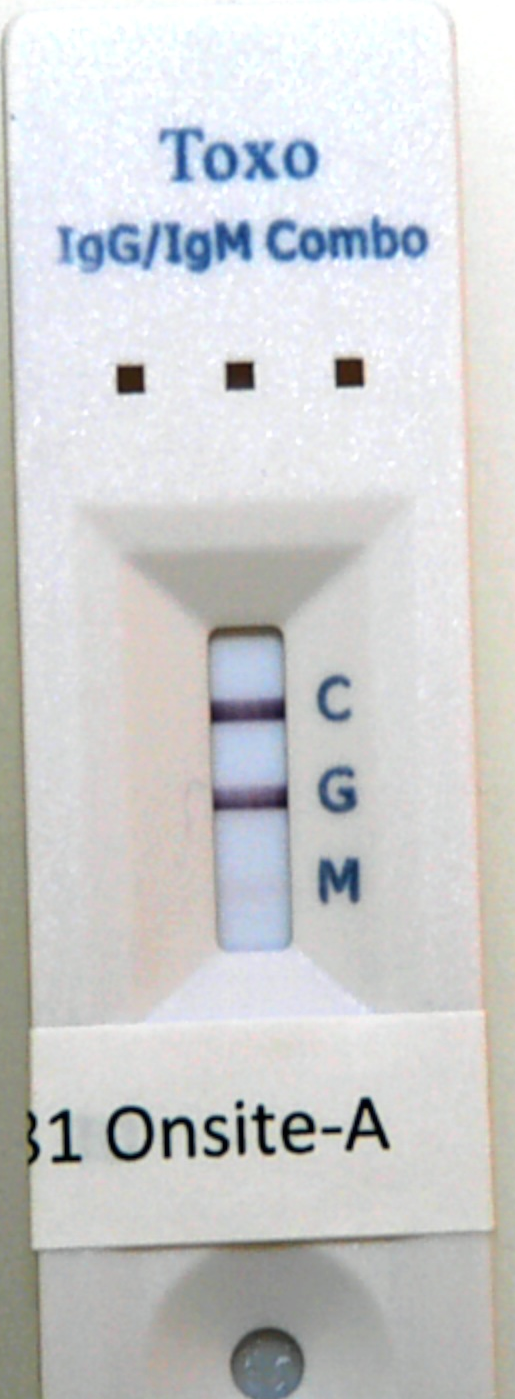

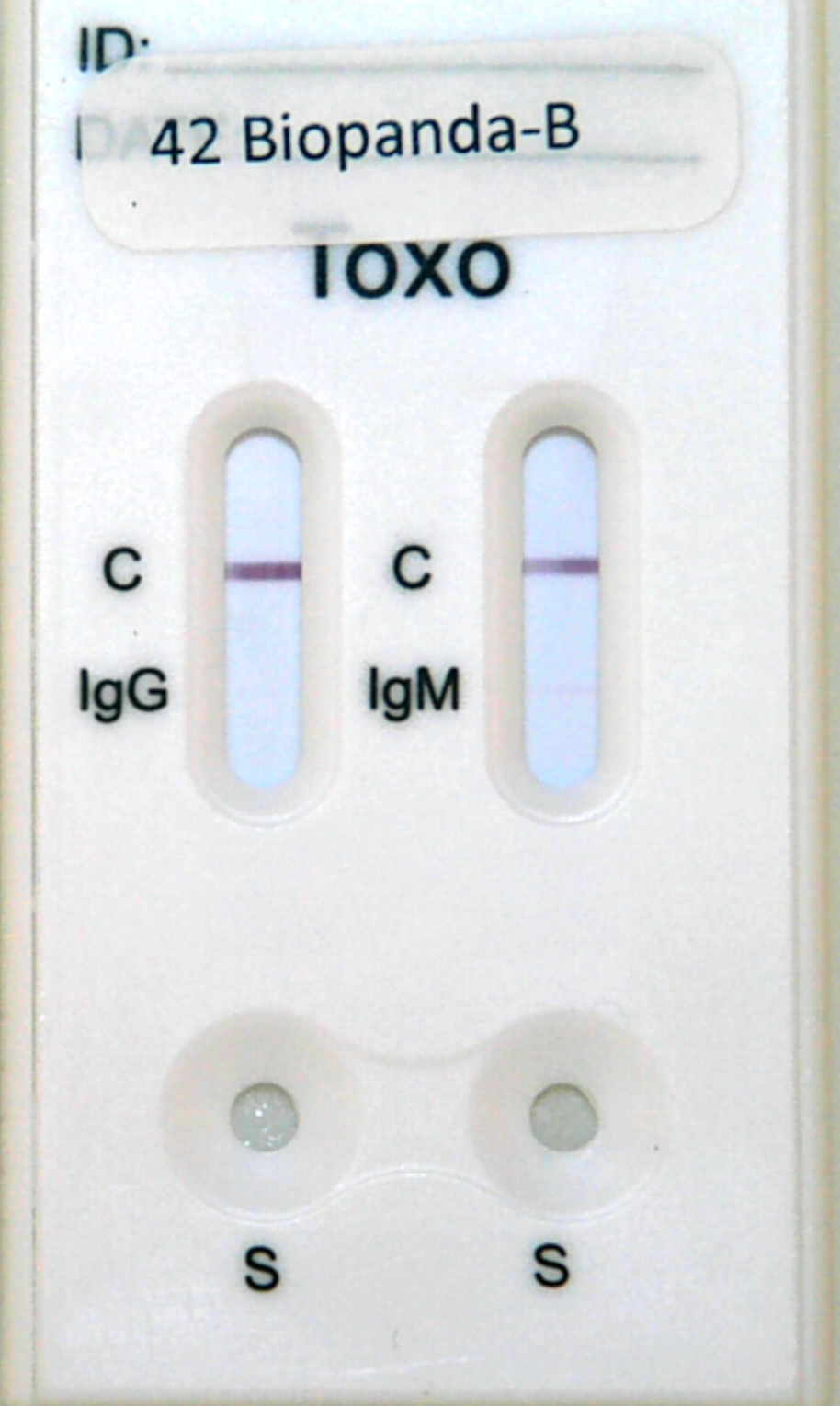


**References**

1. Montoya JG. Laboratory diagnosis of Toxoplasma gondii infection and toxoplasmosis. J Infect Dis **2002**; 185 Suppl 1: S73-82.
